# Impact of COVID-19 Pandemic on Mental Health and Socioeconomic Aspects in Greece

**DOI:** 10.3390/ijerph20031843

**Published:** 2023-01-19

**Authors:** Tasos Stylianou, Konstantinos Ntelas

**Affiliations:** 1 Business Administration, School of Social Sciences, Hellenic Open University, 26335 Patra, Greece; 2 Big Data Analytics, School of Computing, Mediterranean College of Thessaloniki, 54625 Thessaloniki, Greece

**Keywords:** pandemic, lockdown, mental health, visualization, predictive models, GBM, k-means, clusters

## Abstract

The global outbreak of the COVID-19 pandemic has spread worldwide, affecting almost all countries and territories. COVID-19 continues to impact various spheres of our life, such as the economy, industries, global market, agriculture, human health, health care, and many others. The aim of this study was to investigate the impact of the COVID-lockdowns on people’s mental health in Greece. A descriptive, cross-sectional study was conducted in several urban, semi-urban and rural areas. The survey of 252 Greek people was conducted in spring 2022, and 46.8% of them were female and the other 53.2% were male. Ages were between 19 and 60 years old. Some of the main findings were that most of the participants feel their mental health got worse than before (about 80%), participants with kids were more affected than those who did not have any kids because they had bigger responsibilities and the pandemic might have caused them a lot of problems to deal with. The higher the income, the less they are affected, and people whose jobs did not change dramatically were also less likely to not be much mentally affected. Moreover, the percentage of smokers whose mental health became worse was greater than that among those who did not smoke. The same happened with those who consumed alcohol. Finally, we used the GBM algorithm to find three important predictors and we applied k-means to have a clear picture of the different clusters and how a number of participants are connected according to their answers.

## 1. Introduction

People’s lives underwent tremendous alteration as a result of the COVID-19 pandemic. There were extensive closures of educational, workplace, commercial, and healthcare organizations as a result of public health measures to manage the illness. Children were unable to attend school for protracted periods of time as a result of the switch by many people to working from home. Orders to “stay at home” discouraged people from interacting with their friends, relatives, and neighbors on a daily basis. Access to primary and specialized care has been hampered by healthcare systems’ redeployment of resources to support acute inpatient treatment. The availability of support has decreased as the remaining healthcare services have switched to telemedicine [1].

The public’s levels of depression and anxiety have increased as a result of the disruption of daily routines and the limited availability of social support [2]. When compared to the general population, people who were experiencing more hardship, such as those who lost their jobs, were thought to be at high risk of contracting a severe COVID-19 infection, or had pre-existing mental health conditions, frequently displayed higher levels of depression and anxiety [3,4,5].

The global outbreak of the COVID-19 pandemic has spread worldwide, affecting almost all countries and territories. The outbreak was first identified in December 2019 in Wuhan, China. The phrases endemic, outbreak, epidemic, and pandemic describe how prevalent an illness is now in comparison to its prevalence in the past. Infections are frequently characterized using the phrases endemic, outbreak, epidemic, and pandemic, but other illnesses, including cancer, violence, and hypertension, can also be defined in the same ways [6]. These classifications are generally based on the number of cases of a condition compared to the anticipated number of cases during a specific period of time, as well as how far the cases have dispersed geographically [6,7].

An endemic ailment affects a population at a very predictable, consistent pace and the observed and predicted numbers of cases are almost equal [6]. An outbreak occurs when there is an unexpected increase in the number of persons with a condition. Either there are more cases of an endemic ailment than predicted, or the condition is discovered in a new location. Therefore, a single occurrence can trigger an epidemic [6]. Outbreaks occur in very small areas. An epidemic is a wide-scale outbreak that spreads across a vast geographical region. A pandemic is an epidemic that spreads over the globe. The most well-known instance is the Spanish influenza of 1918, which affected more than one-third of the global population and claimed the lives of almost 50 million people [6].

Even if the term “pandemic” has no universally agreed-upon meaning, it may still be useful to analyze diseases that are frequently referred to as pandemics and to try to understand them better by comparing and contrasting them. Diseases that we may think about were selected experimentally to reflect a range of etiologies, diffusion modes, and emergence eras. They include the severe acute respiratory syndrome (SARS), scabies, West Nile illness, AIDS, cholera, dengue, influenza, plague, acute hemorrhagic conjunctivitis (AHC), and obesity [7]. Is it feasible to pinpoint crucial characteristics that apply to all or almost all of these pandemic diseases? In what fundamental ways are these diseases similar and different?

First of all, we have a broad geographic range. The term “pandemic” is almost always used to describe an illness that spreads rapidly throughout a wide region, such as the cholera epidemic of the 19th century or the human immunodeficiency virus (HIV)/AIDS epidemic of the 1980s. In a recent assessment of the history of pandemic influenza, pandemics were divided into three categories: global, interregional, and transregional (affecting nearby and nonadjacent parts of the world) [7].

The next common characteristic is the Disease transmission. Most applications of the term “pandemic” indicate that disease movement or spread by transmission that can be tracked from place to place, as has historically been done for millennia, in addition to geographic extension (e.g., the Black Death). Moving on, we can see that we have high attack rates and explosiveness. Notorious pandemics have a tendency to have several cases showing up quickly in addition to high attack rates and “explosive” dissemination. This epidemiological characteristic characterizes both common-source acquisition and highly infectious illnesses with brief incubation periods, such as cholera in 1831–1832 [8].

One other common characteristic is novelty. Most frequently, illnesses that are unique or at least connected to novel variations of existing organisms have been described as pandemics, e.g., the appearance of HIV/AIDS when it was first identified in the early 1980s.

Finally, some other common characteristics are infectiousness, contagiousness and severity. The word “pandemic” is less frequently used to characterize potentially non-contagious conditions such as obesity or risky habits such as smoking cigarettes, which are geographically widespread and may be increasing in prevalence globally but are not contagious. Such usage of the phrase often occurs more frequently in public health communication and education than in scientific discourse, indicating an attempt to emphasize the significance of the health problem by using the term pandemic in a colloquial rather than scientific meaning [7]. Some illnesses are occasionally contagious but more frequently spread by alternative mechanisms, such as cholera. Although illness severity has not traditionally been a pandemic requirement, the term pandemic has been used considerably more frequently to describe severe or lethal infection, such as the Black Death, HIV/AIDS, and SARS, than to describe mild diseases.

With the desire to discover what effects the pandemic has on people in Greece in terms of their mental health and income, in our study, we attempt to analyze a questionnaire that was created for that scope. Our emotional, psychological, and social well-being is all part of our mental health. It influences our thoughts, emotions, and behaviors. Additionally, it influences how we respond to stress, interact with people, and make good decisions. Mental wellness is crucial throughout one’s life. This mental wellness of many people was disrupted during the pandemic and the reasons need to be found. We are trying to visualize the results and test them, to find possible correlations between the variables and to apply clustering technique k-means. Identification and classification are crucial because they help us comprehend connections and relationships among objects. In order to predict the outcome of a categorical dependent variable based on one or more continuous or categorical independent variables, a machine learning algorithm is trained on a labeled dataset as part of the supervised learning process covered in this paper. The classification model selected to create a prediction model in our case is a gradient boosting machine (GBM). The current study’s objective was to look at how the COVID-19 pandemic and the level of lockdown affected the mental health of people in Greece. This study attempts to answer questions like: Did people get affected mentally due to the pandemic? In which ways were they affected? Can we create predictive models for mental effects? This study is among the first national sample studies tracking temporal changes in population mental health in the context of the COVID-19 pandemic in Greece.

## 2. Literature Review

The seventh human coronavirus, the severe acute respiratory syndrome coronavirus 2 (SARS-CoV-2), was first identified in December 2019 in Wuhan, Hubei province, China [9,10,11]. Since then, the virus has spread over the whole planet, infecting over 600 million individuals and killing 6.5 million as of 20 August 2022. Middle East respiratory syndrome coronavirus (MERS-CoV), SARS-CoV, and SARS-CoV-2, all produce severe pneumonia with mortality rates of 2.9%, 9.6%, and 36%, respectively [12,13,14]. Scientists have argued regarding the origin of the new coronavirus SARS-CoV-2 ever since its discovery [15]. It has been hypothesized that SARS-CoV-2 was created by manipulations in a lab. Genetic evidence, on the other hand, refutes this theory and demonstrates that SARS-CoV-2 did not originate from a previously identified viral backbone [16].

The psychological responses of the population during an infectious illness outbreak have a significant impact on the disease propagation as well as the occurrence of emotional distress and social disorder both during and after the outbreak. Despite this, adequate resources are frequently lacking to control or lessen the negative consequences of pandemics on mental health and wellbeing [17]. The acute period of an outbreak may make this comprehensible, but as health services focus on testing, stopping the spread of the disease, and crucial patient care, psychological and psychiatric needs should not be neglected at any point in the pandemic management process.

This is due to a variety of factors. It is well recognized that psychological variables have a significant impact on how people adhere to public health interventions (e.g., vaccination) and deal with the risk of infection and the ensuing losses [17]. These are unquestionably essential factors to take into account when managing any infectious condition, including Covid-19. Maladaptive behaviors, emotional distress, and defensive responses are examples of psychological responses to pandemics. Those who are predisposed to psychological issues are particularly at risk.

Increased stress and conflict among households may also result from physical separation and social isolation. Quarantined individuals may experience agitation, rage, sleeplessness, anxiety, and sadness [18]. Indigenous groups could be more vulnerable, especially if they reside in isolated locations with inadequate access to and acceptance of services. Protecting indigenous communities need to be a top priority given the history of pandemic exposure among indigenous communities. Marginalized groups may lack adequate living conditions, access to basic commodities, or knowledge of how to maintain personal hygiene. As a result of potentially unstable housing conditions, increased stigma, discrimination, and limitations on their freedom of movement, immigrants and refugees will be especially vulnerable during the epidemic.

Quantifying the pandemic’s effect on mental health presents significant methodological difficulties. According to studies, many participants believed their mental health had gotten worse during the pandemic’s breakout [19,20], with studies of both UK and Chinese individuals showing an increase in the frequency of self-reported experiences of despair and anxiety. However, reports of changes in mental health that are made in the past are highly biased [21,22].

In contrast to cohorts that completed assessments prior to the pandemic, other research has discovered a higher incidence of mental health disorders in cohorts recruited during the pandemic [23,24]. For instance, in samples taken during the early stages of the pandemic compared to samples taken before the beginning of the pandemic, both distress [23] and depression [24] were enhanced in US adults. However, differences in sample recruitment methods, such as a greater reliance on online and non-probability-based samples during the pandemic, and variations in the demographic profiles of cohorts before and after the outbreak make it difficult to infer changes in mental health that can be attributed to the pandemic [25].

In order to assess how mental health has evolved, some longitudinal cohort studies have taken samples from the same patients both before and during the pandemic. Daly et al. [24] discovered that non-specific general mental health symptoms rose in April to June 2020 compared to a pre-pandemic baseline in a sizable nationally representative sample of UK individuals. Other long-term cohort studies have discovered inconsistent or little change in mental health [26,27]. The COVID-19 pandemic’s effects on mental health have a time course that can only be described by longitudinal investigations. A recent multi-wave longitudinal study of US adults found that after an initial rise in distress during the early stages of the pandemic, distress decreased to pre-pandemic levels within a few months, despite the possibility that the effects of the pandemic on mental health could be long-lasting [28].

All facets of our life have been impacted by the epidemic and the societal response that followed in an effort to stop the virus’s spread. The widespread shutdown of educational institutions as well as “non-essential” workplaces and companies, e.g., leisure and entertainment venues, fitness centers, and community organizations, was a result of public health instructions to socially isolate and prevent gatherings. Locally issued “stay at home” orders forced people to stay in their homes for stretches of time ranging from weeks to months in an effort to slow the rate of COVID transmission. Due to their inability to run into friends or relatives at social gatherings, many people suffer from social isolation and loneliness [29].

The pandemic’s effects on the economy are extensive. Due to these interruptions, there were many job losses and little employment options, which significantly increased financial stress [30]. Those who are able to work from home may have difficulties because of the added burden of juggling a job and supporting dependent children in their homeschooling [31]. The majority of the economy was shut down as a social reaction to COVID-19, which in turn caused unexpected, rapid, huge unemployment. Due to their incapacity to effectively lobby for such aid, poor and/or non-democratic countries would be disproportionately affected by the unexpected arrival of widespread poverty in the absence of offsetting economic measures. Economic measures are being implemented in many developed countries, but problems arising from the difficulty of putting such significant subsidies into effect would leave many people vulnerable to poverty and its negative effects on their mental health, such as anxiety and depression [32,33].

The increased symptoms of depression and anxiety are the most typical ways that an excess of stresses and a lack of supportive resources can damage mental health. The prevalence of sadness and anxiety has increased during the pandemic compared to pre-pandemic samples, according to a variety of community-based surveys. One survey, for instance, indicated that 17% vs. 5% of males and 22% vs. 9% of women reported having moderate to severe depressive symptoms during as opposed to before the pandemic [2], and another found that the prevalence of depressed symptoms had increased by more than three times [34]. According to research that took into account individual differences, people who classified as being in a COVID-19 risk category had greater levels of depression and anxiety [4]. Additionally, people who suffered more severe disruptions in their daily lives, such as losing their jobs, were also more likely to have poor mental health [5]. Last but not least, more women and those with pre-existing mental health disorders than the general population reported poorer mental health during the pandemic [3].

The COVID-19 pandemic has brought into focus the mental health of various affected populations. It is known that the prevalence of epidemics accentuates or creates new stressors, including fear and worry for oneself or loved ones, constraints on physical movement and social activities due to quarantine, and sudden and radical lifestyle changes. Governments all across the world have enforced their version of mandatory self-isolation through the implementation of lockdown measures in an effort to stop the spread of COVID-19 and minimize the loss of life. Unfortunately, limiting people’s freedom of movement and denying them of their most important possessions may exacerbate the detrimental impact on happiness levels. This may be magnified in an extreme rural situation. An extreme country is defined as one that has extremely severe lockdown laws, high infection rates, and low levels of wellbeing. We define well-being as those facets of life that society as a whole recognizes as critical to a person’s welfare, happiness, and quality of life. Material (income), one of the well-being components, is dependent on a dismal economic outlook [35].

Some new research papers introduce new techniques to find the consequences of COVID-19, such as the novel machine learning-based COVID-19 detection framework, multilayer network-based approach, and Valence Aware Dictionary and Sentiment Reasoner (VADER) [36,37,38].

### The Case of Greece

On 26 February 2020, the first verified COVID-19 case was detected in Greece and. as a result, all schools were closed. Cafés, pubs, museums, shopping malls, sports facilities, and restaurants were shuttered on 13 March; one day after the first fatality from COVID-19 was reported. Retail stores followed on March 16. Finally, on March 23, restrictions on all non-essential movement across the nation went into effect. All movements outside the home for specific reasons, such as health reasons, purchases of necessities, helping others in need, and exercising, now require a signed attestation or mobile phone SMS notification. After a 42-day lockdown, restrictions were progressively relaxed beginning on 4 May. A total of 2632 confirmed cases and 146 fatalities were reported up to that point. In August, the second wave of the pandemic started, and by mid-October, it intensified much more. From a maximum of five fatalities per day in the spring during the first wave of the pandemic, the number of daily deaths began to grow in mid-September and continued to rise by early November, reaching a seven-day moving average of 34 deaths per day (on 11 November 2020). On that day, the second strict lockdown was announced. Schools, shops, restaurants, and nightclubs were closed, and only a limited number of exceptions, such as those related to work, health, exercise, purchasing food supplies, and helping those in need, were permitted to leave the house without a written attestation or mobile phone SMS notification [39]. It became necessary to present a certificate of full vaccination (two or three doses up to nine months from the last dose), a disease certificate (up to 180 days from the positive result), a negative 72-h PCR test result, or a rapid test of 48 h during the third and final restriction period, which began on 6 November 2021, and ended on 30 April 2022. For all indoor venues with the exception of supermarkets, pharmacies, and bakeries, it was sufficient to show a negative self-diagnostic test for children up to the age of 17.

Greece was able to limit the pandemic during the initial spring wave, recording comparatively few confirmed COVID-19 cases and fatalities compared to other nations [40]. The nature, length, and severity of the enforced restrictions, however, were the same as those imposed by other nations where the epidemic had taken a less positive course. Furthermore, the Greek healthcare system has just lately begun to recover from a severe budgetary crisis that had a significant social impact. Budget cuts for hospitals in recent years have resulted in understaffing, sporadic medical supply shortages, and restricted access to treatment and preventative treatments [41]. Despite increasing demand for mental health services during public health crises [42,43,44], these services typically experience significant budget cuts because, in contrast to services focusing on physical health, they frequently lack a strong lobbying base to defend their importance. Services for children’s mental health appear to be particularly susceptible to budget cuts. Major funding modifications have been made to Greece’s child and adolescent mental health services and supportive policies. The number of abused or neglected children admitted for child protection to pediatric hospitals increased dramatically, while many non-profit child and adolescent mental health community centers, psychosocial rehabilitation units, and highly specialized establishments were suspended [45] as a result of public funding cuts.

The COVID-19 pandemic and the ensuing lockdown measures are anticipated to have a significant and perhaps long-lasting impact on mental health, particularly among the most vulnerable. Almost all citizens are expected to be particularly affected [46,47,48,49,50,51,52,53]. Increases in posttraumatic stress disorder (PTSD), depression, anxiety, drug use disorder, sleep difficulties, numerous other mental and behavioral diseases, marital violence, and child abuse nearly usually follow major catastrophes [54]. Such occurrences can have an adverse effect on one’s mental health both immediately and for some time afterward. For instance, increases in PTSD, anxiety, and general psychological distress were linked to the SARS pandemic in 2003 in both patients and healthcare workers [55]. There is a dearth of pertinent literature examining the sort of psychopathology that develops in populations of children and adolescents following major events [56]. The most common psychological symptoms displayed by kids and teens of all ages during the COVID-19 outbreak were clinging, inattention, and irritability, according to published data from China, the first country to be hit by the novel coronavirus [56].

Early concerns about the virus centered on respiratory failure, but a rapidly expanding body of research suggests that COVID-19 may have broader effects than first thought. In particular, the majority of people in the world experienced disruptions to daily life brought on by public-health measures taken to curb the spread of COVID-19 during the years of the pandemic, which may have created psychological difficulties.

In sum, in our study, we attempt to examine the effects that the pandemic has had on mental health. Three research questions are explored: 1. Did the crisis negatively affect the mental health of Greek adult people? 2. Is there any significant model which can examine the relationship of mental health with other quantitative predictor variables? 3. Is there any connection between age and mental health issues due to the pandemic?

## 3. Materials and Methods

With the desire to discover what effects the pandemic has on people in Greece in terms of their mental health and income, in our study, we attempt to analyze the questionnaire that was created for that scope. We have collected 252 questionnaires from all parts of Greece and from different ages, males and females. The data were collected during spring 2022. We are trying to figure out the descriptive statistics of the dataset, to search for possible correlations between the variables and the last part is the classification and evaluation of the applied model. The assumptions, methods, and implementation utilized in this study are acceptable for cluster-based distributed deployment architectures, even if performed on local resources. For statistical computation and graphics, R is employed. A pilot study was carried out in a sample of 40 subjects before the final study. The questionnaire contained four parts: (1) title, summary, and consent form, (2) sociodemographic information, (3) questions regarding measures against COVID-19 and mental health. The respondents had to fill out the self-reporting questionnaire and had the liberty of dropping out of the survey at any time. The proposed methodology is applied for first time for the country of Greece during the COVID-19 pandemic times.

### 3.1. Classification (Supervised Learning)

Numerous applications of big data analytics and machine learning (ML) have been described recently, with a focus on the classification issues and techniques. The basic goal of classification algorithms is to develop a model that, in theory, generates the same labeling for the supplied data and performs well on untried data (i.e., prediction) [57]. A classification issue is the process of predicting the label of a K-dimensional input vector x, where $xa??XaS?R^{k}$ (keep in mind that most ML algorithms need real-valued input variables) and $y\to Y=\left\{C_{1},C_{2},\ldots ,C_{Q}\right\}$. A classification rule or function g: X→Y that can forecast the label of novel patterns is used to complete this research. In the supervised context, we are provided with a training set of N points, denoted by D, from which g will be modified, D = {(xi, yi), i = 1,..., N} [57].

The classification model’s prediction performance is evaluated using different standards depending on the problem being studied, the type of data being used, and the classifier’s intended use. The following evaluation measures are used to assess categorization performance.

#### 3.1.1. Confusion Matrix

The classification performance of a classifier in relation to certain test or validation data is summarized by a confusion matrix [58]. The confusion matrix shows a summary of all prediction results, with both right and bad guesses having one of four possible outcomes:

- Real positive (TP). The actual value is in line with the expected value. The model anticipated a positive value, and the actual result was positive.
- False positive (TN). The actual value is in line with the expected value. Although the model had anticipated a negative result, the actual value was negative.
- Inaccurate positive (FP). The value was incorrectly anticipated. The model anticipated a positive result, but the actual value was negative.
- Deceptive negative (FN). The value was incorrectly anticipated. The model projected a negative result, while the actual value was positive.

Based on the confusion matrix, a number of classification performance metrics are computed, including the following:

- The accuracy is determined by dividing the total number of observations by the total number of positive and negative outcomes of all right predictions.
- The number of correctly anticipated positive outcomes divided by the total number of real positives is used to compute the true positive rate, sensitivity.
- Specificity is determined by the number of correctly anticipated negative outcomes divided by the total number of actual negatives.
- To determine precision, divide the total number of positive predictions by the proportion of correct positive predictions (true and false).

#### 3.1.2. Receiver Operating Characteristics Curve (ROC)

The ROC curve illustrates the trade-off between the true positive rate (TPR) and false positive rate (FPR) metrics for a classifier under various decision thresholds by plotting TPR on the y axis versus FPR on the y axis. Higher values suggest greater prediction performance [59]. The area under the ROC curve is known as the area under curve (AUC) [60] and is a measure of the model’s capacity to determine class labels. AUC values vary from 0.5 to 1, with a value of 0.5 being considered random guessing-level predictability and a value of 1 being regarded the highest predictability [60].

#### 3.1.3. K-Means

The K-means algorithm was also applied in our dataset. The flat clustering algorithm is yet another name for it. The letter “K” in K-means stands for the number of clusters that the algorithm identified from the data. According to this strategy, data points are grouped into clusters so that the total of their squared distances from the centroid is as little as it can be. In other words, the K-means method finds k centroids and then assigns each data point to the closest cluster while minimizing the size of the centroids [61].

### 3.2. Data Ethical Concerns

All actions were taken in accordance with the Policy and Code of Practice on Research Ethics [62], taking into account the significance of producing a valid result, safeguarding the privacy and improper handling of the data available, and protecting the integrity of the person. The questionnaire is designed to gather information and those who participated in this research, participated completely voluntary. All of the responses were recorded anonymously and will remain private. Participants have the right to withdraw their participation at any time without any consequence. Lastly, every participant had to be 18 years old or more.

### 3.3. Design of the Questionnaire

In order to create the questionnaire, factors that have effects on mental health were researched. Genetics, which is sometimes an important factor, were not taken into account as we try to focus more on the results of the pandemic.

- Social connections: Interactions with other individuals have a significant role in our daily lives. We are prone to experience grief-related feelings if we have a quarrel or lose one of our connections. Even when we are surrounded by people, loneliness can have a negative effect on our mental health. It is more difficult to overcome our emotions of loneliness because of the present epidemic and the resulting social constraints [63].
- Housing and money: Anxiety about our employment, housing, or financial condition might harm our mental health. Our feeling of purpose might be shaken while we’re unemployed, and it could be challenging to keep our confidence up. Our mental health may be impacted by problems with landlords, home repairs, or making mortgage payments [63].
- Physical well-being: Health issues, chronic illnesses, life-threatening illnesses, doctor’s visits, and tests may all negatively impact our mental well-being and cause us to feel nervous and unhappy [63]. There are many people who are concerned about their health as a result of the current COVID-19 outbreak.
- Addiction and drug abuse: Alcohol and tobacco abuse can have a negative impact on mental health. Addictive behaviors and increasing drug abuse can both be caused by poor mental health. This can get out of control [64].

### 3.4. Dataset Description

Our dataset contains 252 rows (total number of answers) and 22 variables. The 22 variables (Table 1) are the answers to the questionnaire questions.

### 3.5. Dataset Preprocessing and Demographic Data

In our dataset, we have set as a rule for ethical reasons that everyone who participates has to be over 18. It is also appropriate to change the type of most of our variables as they were recognized as characters. We prefer to use numeric and factors because in this way the data analysis will be more effective. Finally, we had to deal with some missing values and one of the best possible ways to deal with this problem is to replace them by mean of every column [65]. We collected 252 questionnaires in order to investigate the effects of the pandemic on mental health. Among the 252 respondents, 118 (46.8%) were female and the other 134 (53.2%) were male. Their ages were between 19 and 60 years old. Further, 111 of them were single, 95 married, 33 divorced, 5 windowed, and 8 of them of other status. Finally, according to their educational status, 95 of them hold a bachelor’s degree, 63 a master’s degree, 57 are high school graduates, and 18 of other status.

## 4. Analysis and Discussion

In this section the results of our data analysis will be presented and discussed. First of all, the descriptive statistics will be given so that we can get some important insights of the dataset and get some first results. After that, statistical analysis will be applied to specific variables that have unusual results and need to be further investigated. Finally, we will use gradient boosting machine (GBM), a boosting algorithm to create a prediction model and we will evaluate it.

### 4.1. Descriptive Statistics

In Table 2, we present the descriptive statistics for all the variables (numeric and categorical). 

The ages of the participants are well balanced overall, and that is extremely important for our results as it means that our sample is good and will get representative results. The question regarding the frequency of meeting friends during the pandemic was: “How often were you able to see your friends during the pandemic? (Scale 0 to 100, 50 means no change, 0 means I couldn’t meet them at all, 100 means I was able to see them way more than before)”. Most of the participants met their friends less than what they used to. This is an important indicator that they might got worsen in their mental health [46]. Regarding the insecurity level the question was: “Did pandemic made you feel more insecure than before or not (scale 0 to 100, 50 means no real change, 0 means way more confident than before, 100 means way more insecurity than before)”. Most of the participants felt way more insecure than before. This is also an indicator that they are mentally affected negatively [63].

The question about work pressure was: “How much more or less pressure did you felt in your work environment during the pandemic (scale 0 to 100, 50 means no real change, 0 means no pressure at all, 100 means a lot more pressure)”. It seems that most of them, feel the same as before but also an important part feels more pressure than before. Only a few persons reported feeling less pressure. Finally, it is worthy to say that for the question “Did you have psychosomatic symptoms during the pandemic?”, 79.8% answered yes and only 20.2% answered no.

The question about the affection of mental health was: “Our emotional, psychological, and social well-being is all part of our mental health. In which way do you think that pandemic and all the restrictions have affected your mental health?” The results here were impressive because 80.2% of the responders answered that the restrictions have worsened their mental health. In Table 3, we will present the variable of mental health versus some other variables. It is also necessary to test them with the chi square test, to confirm or not if there is strong relationship between them. 

To summarize the results of Table 3, we can say that, in Greece, most of the participants feel that their mental health got worse than before, approximately 80%. Although, it was not expected that the gender would be an important factor [63], the percentage of women whose mental health worsened is 86% while that of men is 75%. This will be further investigated later in the statistical analysis. Moreover, participants with kids were more affected than those who did not have any kids. This was also expected because they had bigger responsibilities and the pandemic might have caused them a lot of problems to deal with.

Furthermore, we can see that the higher the income the less they are effected, which was also expected [66]. Participants with higher salaries were not affected as much as those who had lower incomes [63]. Regarding the variable job-affected mental health (the question was “How much has the pandemic affected your job?”) we found that people whose jobs did not change dramatically were also less likely to not become much mentally affected. This result was also expected according to previous research [63]. It is also noticed that participants with a higher level of education were less affected by the pandemic. This also has to do with their ability to find easier a job [67]. Participants who were able to travel also did not feel this much worse in terms of their mental health. People who were not able to travel feel way more depressed [68] so it was also an expected result. Finally, the percentage of smokers whose mental health got worse is bigger than that among those who did not smoke, which was also a result that was expected, as smoking causes mental health issues [64].

Furthermore, we can see the connection between the variable restrictions (The question was “Did you feel that government’s restrictions during the pandemic have reduced your freedom?”) and mental health. Participants who felt that the government did not reduce their freedom feel the same or better than before, i.e., 100%, which is a very impressive result. On the other hand, people who felt that they weren’t free mostly had their mental health got worse. Generally, when someone does not feel free, they are more likely to have mental issues [63].

The next pair represents the connection between the variable alcohol (The question was “Do you consume alcohol?”) and mental health. Moreover, just like smoking, alcohol, can cause mental health issues or make them get worse [64]. This is shown in our table, as the percentage of those who do not consume alcohol whose mental health became worse is way lower than those who consume alcohol.

Finally, the last pair of variables refers to the connection between marital status and mental health. It seems that people who are not married and tend to have fewer responsibilities to other people presented a lower percentage of mental health reduction.

### 4.2. Gender and Mental Health

The results between gender and mental health need to be investigated further. A chi-square test is used to help determine if observed results are in line with expected results, and to rule out that observations are due to chance (Table 4). We have a chi-squared value of 2.6871 and *p*-value 0.1012. Since we get a *p*-value greater than the significance level of 0.05, we accept the null hypothesis and conclude that there is no significant relationship between the two variables [69]. So, although there was difference in the percentages of Table 3, there is no significant relationship between gender and mental health. That means that there is no relationship between gender and mental health (that is, if they are independent). Then, the actual frequencies at which male and female participants’ mental health was affected should be expected to be approximately equal, or conversely, the proportion of male and female people in Greece course should be approximately equal to the proportion of male and female participants in the sample. In order to make this even more solid, it was also tested with Fisher’s exact test and a *p*-value of 0.1082 was obtained, which confirms the chi-square test results (Table 4).

#### Age and Mental Health

**Figure 1:**
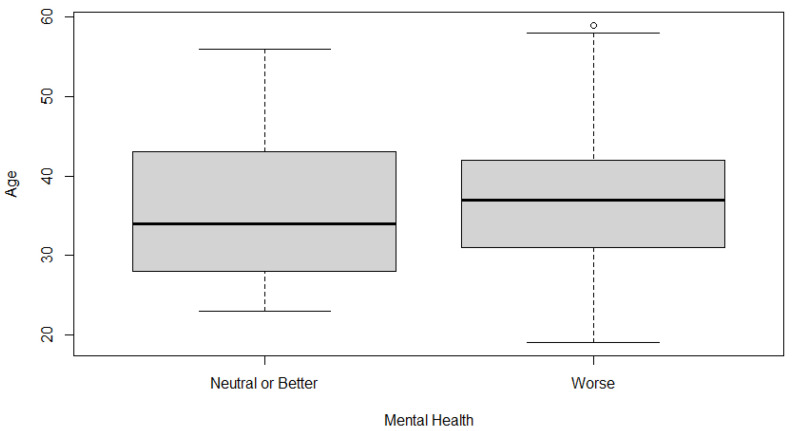
Boxplot of mental health different answers according to age.

Although according to the bibliography there is no connection between age and mental health issues due to the pandemic [46], it remains necessary to investigate in depth. An independent samples t-test is performed to test for differences in means in age between the two different answers in mental health (neutral or better and worse). The variable age holds the age of each participant, and the variable mental health indicates whether a participant’s mental health is worse or neutral/better. As shown in Table 5, the means between the two mental health categories according to age are different with the first being 35.8 and the second 37.32, also being visible in the box plot diagram shown in Figure 1.

Since the samples in the two classes are unrelated, the independence assumption for doing the t-test holds true. Moreover, after using the Shapiro test [69], we get a *p*-value of 0.2196 (Table 6), which indicates that we have a normal distribution. We also use Levene’s test [70], which guarantees that the two groups have equal distribution around the mean to test the homoscedasticity statement. As seen in Table 6, the test yields a *p*-value greater than 0.05, meaning that the group variances are not significantly different.

Using the t-test (Table 7) the *p*-value is 0.3919, which is significantly higher than the 0.05 significance amount. Since the *p*-value is slightly higher than 0.05, we conclude that the variance in means is more likely due to chance, so we do not dismiss the null hypothesis. The 95% confidence interval means that the population difference is between negative 5.062784 and positive 2.023440, 95 times out of 100. In this case, we can conclude that for the observations included in this dataset, both “worse” and “neutral or better” participants’ age mean in years has no significant difference, with *p* value of 0.3919.

### 4.3. Multi Linear Model

A multiple linear regression model [71] was created to examine the relationship of the quantitative variable mental health, with a range of other quantitative predictor variables. The regression model was then validated against assumptions of linearity and multicollinearity. The variable mental health is dichotomous and is converted to one and zero. The insecure variable is first added in the model. This variable indicates whether a participant felt more insecure or not because of the pandemic. This one supposed to be one of the most important (The question was “Did the pandemic make you feel more insecure than before or not (scale 0 to 100, 50 means no real change, 0 means way more confident than before, 100 means way more insecurity than before)). We then add to our model some more components, e.g., friends (the question was “How often were you able to see your friends during the pandemic? (Scale 0–100, 50 means no change, 0 means I could not meet them at all, 100 means I was able to see them way more than before)) and work pressure (The question was “How much more or less pressure did you feel in your work environment during the pandemic (scale 0–100, 50 means no real change, 0 means no pressure at all, 100 means a lot more pressure)).

So, our estimated model is:

$$MH=b_{0}+b_{1}INS+b_{2}FR+b_{3}WP+e_{i}$$

where: MH is mental health, INS the variable of insecure, FR for the variable friends, and finally WP for the variable work pressure. The results are presented in Table 8.

The empirical outcomes of the study reveal that the sign of insecure variable is negative and its *p*-value is (0.000), which means it is highly significant at 1% level. The variable of friends is positive, and it is significant at the 5% level. Finally, the variable of work pressure is negative, and it is significant at the 10% level. The R-squared value is 0.236 and adjusted R-squared is 0.218.

The variance inflation factor (VIF) (Table 9) is used to test the multicollinearity assumption or the scenario where independent variables in the model are strongly correlated to each other. All predictor VIF values are near 1, meaning that multicollinearity is not present.

### 4.4. Generalized Boosting Model and Evaluation

In order to construct a classification model and forecast the class of a dependent binary variable (variable mental health in the dataset) which indicates whether a participant is worse or neutral/better, the gradient boosting machine (GBM) algorithm is used [72]. Data are arbitrarily divided between training and a test set in an 80:20 ratio for training and evaluating the model. The predictive power of a classification algorithm is typically measured by its accuracy (or error rate, which is 1 minus the accuracy). In addition to probability estimations 1 or “confidence” in class prediction, most classifiers will produce them. That is, the probability parameter ignores the uncertainty of the forecast (which can range from 0.51 to 0.99); it is called reliable if the class of the highest chance prediction is the same as the target. Although the true probability of the study examples is unknown, it is often assumed [60]. The confusion matrix output will be used to test the GBM model’s prediction accuracy and area under curve (AUC) [60].

To find the best possible predictors, we first try a model with every variable and use a summary of the model so that we keep only the important features. By fitting the model, we found three variable predictors (anxiety, insecurity, and work pressure), and all of our predictors are significantly associated to the outcome. To get the best possible cut, we use receiver operating characteristics curve (ROC).

According to our results, the area under curve (AUC) is almost 0.95, which indicates an excellent model [60]. On the other hand, evaluating with confusion matrix (evaluation with accuracy, sensitivity, precision, F-measure, and specificity), we have a very good accuracy, more than 0.9, and every other measure is over 0.82, indicating a very good working model. As shown in Table 10, the overall evaluation of the model is pretty good.

### 4.5. K-Means

Finding the centroid is what “means” in the K-means analysis refers to when averaging the data. Our aim is to gain some very important information about the connection between insecurity and work pressure which are undoubtedly two of our most important variables. Insecurity is an extremely important factor for mental health. A person who generally has less work pressure generally tends to have less anxiety, so it is a parameter that cannot be ignored. We investigated the connection of these two. Firstly, we found with the ankle rule that the best cut is K = 4 (the optimal k is the point where the curve is starting to have a diminishing return), and then we performed our clustering. The result was that we can divide participants into four different categories: participants that had lower insecurity and lower work pressure during the pandemic, those who had almost same level of insecurity and work pressure like before, those who had same level of work pressure but increased insecurity (by other factors), and lastly, those who had increased insecurity and work pressure. From this visualization, we can observe that these two are pretty much connected and correlated and most of the participants are in cluster 3.

## 5. Conclusions

When considered together, the available data show that the ongoing COVID-19 pandemic is seriously affecting people’s mental health. During the early stages of the COVID-19 pandemic, people suffered from significant psychological distress, including anxiety, sadness, and post-traumatic symptoms. This study is among the first national sample studies to track temporal changes in population mental health in the context of the COVID-19 pandemic in Greece.

Overall, the intensity of the findings was rather consistent: the majority of people had mild-to-moderate disruptions, while those with severe symptoms made up a smaller percentage of the population. Some of the main findings were that most of the participants felt their mental health got worse than before (approximately 80%). Participants with kids were more affected than those who did not have any kids and that was expected because they had bigger responsibilities and the pandemic might have caused them a lot of problems to deal with. Moreover, the higher the income, the less they are affected, which was also expected, and participants with higher salaries were not as affected as those who had lower incomes. People whose jobs did not change dramatically were also less likely to not be much mentally affected and participants with higher level of education were less affected by the pandemic. This has to do with their ability to find easier a job. The participants who were able to travel also did not feel this much worse in terms of their mental health. On the other hand, those who were not able to travel felt way more depressed. Finally, the percentage of smokers whose mental health got worse is bigger than among those who did not smoke, as smoking causes mental health issues. The same happened with those who consumed alcohol.

Further, in the GBM, we arrived at a model that has excellent performance and contains 3 important predictors. The AUC was almost 0.95, which indicates a great model. Finally, applying k-means gave us clear picture of the different clusters and how a number of participants are connected according to their answers in four different clusters.

The lockdowns, self-isolation, social distancing, and quarantine have affected the overall physical, mental, spiritual, and social wellbeing of the Greek people. With the beginning of lockdowns, the government decided to shut down all cinema halls, gyms, health clubs and museums, as well as banned the gathering of people for cultural, social, or religious activities, including temples, monasteries, and churches. Despite the fact that the measures were taken for the protection of people from COVID-19, their result was to create fear, anxiety, and uncertainty among the people and to worsen their mental health. The economic recessions have put significant financial pressure on many families, which might increase unhealthy conflict, family breakdown, abuse, depression, and domestic violence. The impacts of the pandemic might be a challenge for an indefinite time. Hence, it is necessary to emphasize and address coping strategies, mental health interventions, and awareness using the available resources. The governments should and must find a way in future health emergencies to help people to retain their mental health.

### Limitations and Future Work

Although our sample (252) can give us a good general idea of the effect of COVID-19 on mental health, additional extensive trials are needed to offer a more thorough investigation of the effect. The purpose of this study was to demonstrate this effect on mental health with the help of data analytics. Future research could extend the pool of the data and get even better results both in their statistical analysis as well as their predictive models.

Our study is about Greece only and it does not include other countries which had different types of social distancing. On the other hand, to show the connection between the influence of COVID-19 on mental health and cultural behavior, this analysis would be broadened to include additional locations as well as other cultures. Future studies should also focus on the creation of effective preventive, treatment, and recovery plans for a global public health disaster such as a pandemic. Making targeted interventions for the most impacted groups would be another difficulty. Public health and mental health groups are issuing helpful advice on maintaining mental health and wellbeing. The National Alliance on Mental Illness (NAMI), the Substance Abuse and Mental Health Services Administration (SAMHSA), and the American Psychiatric Association (APA) all offer basic advice for the public on how to manage their time and physical and mental health. Further details about the high-risk categories are provided by the WHO and the Centers for Disease Control and Prevention (CDC). Convincing evidence currently points to a connection between the COVID-19 pandemic, lockdown, socioeconomic effects, and mental illness, even if certain fundamental features of these relationships require additional elucidation. It is necessary to look at potential risk and protective variables in more detail.

## Figures and Tables

**Table 1:** Dataset description.

Number	Feature Name	Description/Question
1	gender	Gender of the person (Male/Female)
2	age	Age of the person
3	education	Education level
4	marital	Marital status
5	kids	Do you have kids?
6	kids_number	How many kids do you have? (If you don’t have type 0)
7	job	Job status
8	wage	What is your wage per year? (in euro)
9	exercise	How many times do you exercise per week?
10	smoke	Do you smoke?
11	alcohol	Do you consume alcohol?
12	travel	Were you able to travel during the pandemic?
13	friends	How often were you able to see your friends during the pandemic? (scale 0 to 100, 50 means no change, 0 means I couldn’t meet them at all, 100 means I was able to see them way more than before)
14	job_effect	How much pandemic affected your job?
15	job_way	How did you work during the pandemic
16	income_change	Did pandemic reduced or increased your income? (scale 0 to 100, 50 means no change, 0 means lost my income, 100 means my income increased a lot)
17	anxiety	Did you have bigger anxiety than usual during the pandemic?
18	psychosomatic	Did you have psychosomatic symptoms during the pandemic?
19	insecure	Did pandemic made you feel more insecure than before or not(scale 0 to 100, 50 means no real change, 0 means way more confident than before, 100 means way more insecurity than before)
20	work_pressure	How much more or less pressure did you felt in your work environment during the pandemic (scale 0 to 100, 50 means no real change, 0 means no pressure at all, 100 means a lot more pressure)
21	restrictions	Did you feel that government’s restrictions during the pandemic have reduced your freedom?
22	Effect of mental_health	Our emotional, psychological, and social well-being is all part of our mental health. In which way do you think that pandemic and all the restrictions have affected your mental health?

**Table 2:** Descriptive Statistics.

**Variable Name**		**Frequency**		**Percentage (%)**	
**Gender**					
Female		118		46.8%	
Male		134		53.2%	
**Education**					
PhD		19		7.6%	
Master		63		25.0%	
Bachelor		95		37.7%	
High School		57		22.6%	
Other		18		7.1%	
**Marital Status**					
Married		95		37.7%	
Single		111		44.1%	
Divorced		33		13.1%	
Windowed		5		2.0%	
Other		8		3.1%	
**Kids**					
Yes		124		49.2%	
No		128		50.8%	
**Job**					
Private Employee		106		42.1%	
Freelancer		47		18.6%	
Civil Servant		39		15.5%	
Unemployed		33		13.1%	
Student		14		5.5%	
Other		13		5.2%	
**Wage**					
Less than 12,000 Euro		114		45.2%	
12,000–18,000 Euro		61		24.2%	
18,000–24,000 Euro		37		14.7%	
More than 24,000 Euro		40		15.9%	
**Exercise**					
None		103		40.9%	
Once a week		33		13.1%	
2–3 times a week		61		24.2%	
More than 3 times		55		21.8%	
**Smoke**					
Yes		138		54.8%	
No		114		45.2%	
**Alcohol**					
Yes		181		71.8%	
No		71		28.2%	
**Travel**					
Yes		187		74.2%	
No		65		25.8%	
**Job Effect**					
Not at all		65		25.8%	
A little		86		34.1%	
A lot		101		40.1%	
**Job Way**					
Remote		65		25.8%	
On Site		58		23.0%	
Hybrid		51		20.2%	
Suspended		45		17.9%	
Unemployed		33		13.1%	
**Anxiety**					
Yes		196		77.8%	
No		56		22.2%	
**Psychosomatic**					
Yes		51		20.2%	
No		201		79.8%	
**Restrictions**					
Yes		230		91.3%	
No		22		8.7%	
**Mental Health**					
Neutral or Better		50		19.8%	
Worse		202		80.2%	
**Variable Name**	**Mean**	**Median**	**Min**	**Max**	**Std. Deviation**
**Age**	38.25	37.02	19	60	17.89
**Kids Number**	1.21	0.80	0	5	0.95
**Friends**	42.06	41.18	0	100	32.25
**Income Change**	46.97	50.12	0	100	56.52
**Insecure**	66	65	0	100	83.15
**Work Pressure**	56.32	50	0	100	40.17

**Table 3:** Mental Health vs. some of our variables.

**Variable**	**Neutral or Better**	**Worse**
Mental health	19.8%	80.2%
**Variable**	**Mental Health**	
**Gender**	**Neutral or Better**	**Worse**
Female	14%	86%
Male	25%	75%
X-squared	2.6871	*p*-value: 0.1012
**Kids**	**Neutral or Better**	**Worse**
No kids	25%	75%
Kids	15%	85%
X-squared	2.413	*p*-value: 0.0002
**Wage**	**Neutral or Better**	**Worse**
Less than 12,000	13%	87%
12,000–18,000	19%	81%
18,000–24,000	32%	68%
More than 24,000	29%	71%
X-squared	5.341	*p*-value: 0.0235
**Job Effect**	**Neutral or Better**	**Worse**
Not at all	26%	74%
A little	21%	79%
A lot	15%	85%
**X-squared**	**2.213**	***p*-value: 0.0031**
**Education**	**Neutral or Better**	**Worse**
High School	12%	88%
Master	23%	77%
PhD	19%	81%
Other	0%	100%
X-squared	6.943	*p*-value: 0.0242
**Travel**	**Neutral or Better**	**Worse**
Yes	28%	72%
No	17%	83%
X-squared	2.374	*p*-value: 0.1233
**Smoke**	**Neutral or Better**	**Worse**
Yes	13%	87%
No	25%	75%
X-squared	3.570	*p*-value: 0.0491
**Restrictions**	**Neutral or Better**	**Worse**
Yes	12%	88%
No	100%	100%
X-squared	57.811	*p*-value: 0.0000
**Alcohol**	**Neutral or Better**	**Worse**
Yes	16%	84%
No	30%	70%
X-squared	4.160	*p*-value: 0.0411
**Marital status**	**Neutral or Better**	**Worse**
Single	25%	75%
Married	11%	89%
Divorced	20%	80%
Widowed	67%	33%
Other	20%	80%
X-squared	8.568	*p*-value: 0.0721

**Table 4:** Pearson’s Chi-squared Test and Fisher’s Exact Test for the variable Age.

**Pearson’s Chi-squared Test**					
X-squared	2.6871	Df	1	*p*-value	0.1012
**Fisher’s Exact Test**					
*p*-value			0.1082		

**Table 5:** Descriptive Statistics of the age between the answers in mental health.

Age and Mental Health		
	Neutral or Better	Worse
Min	23	19
Max	56	59
1st Quartile	28.50	31.25
3rd Quartile	42.75	42
Mean	35.80	37.32
Median	34	37

**Table 6:** Shapiro and Levene’s Test.

**Shapiro Normality Test**	
W = 0.98805	*p*-value = 0.2196
**Levene’s Test Homogeneity**	
F Value = 0.5562	*p*-value = 0.457

**Table 7:** Welch Test and Confidence Interval.

**Welch Two Sample *t*-test**	
t = −0.8649	*p*-value = 0.3919
**95 percent Confidence Interval**	
−5.062784	2.023440

**Table 8:** Results of regression model.

Variable	Coefficient	Std. Error	t value	*p*-Value
Intercept	0.7042	0.1527	4.611	0.000 ***
INS	−0.0077	0.0016	−4.806	0.000 ***
FR	0.0029	0.0015	1.985	0.049 **
WP	−0.0022	0.0017	−1.320	0.089 *
R-Squared	0.2336			
Adjusted R-Squared	0.218			

* denotes significant 1%, ** denotes significant 5% and *** denotes significant 10%.

**Table 9:** Variance Inflation Factor (VIF) Test.

Variable	INS	FR	WP
VIF	1.1458	1.1488	1.1873

**Table 10:** Evaluation with accuracy, sensitivity, precision, F-measure and specificity.

Evaluation	
Accuracy	0.903
Sensitivity	0.893
Precision	0.875
F-Measure	0.824
Specificity	0.955

## Data Availability

The datasets generated during and/or analyzed during the current study are available from the corresponding author on reasonable request.
